# Genome Size and Chromosome Number Evaluation of *Astragalus* L. sect. *Hymenostegis* Bunge (Fabaceae)

**DOI:** 10.3390/plants11030435

**Published:** 2022-02-05

**Authors:** Ali Bagheri, Azadeh Akhavan Roofigar, Zahra Nemati, Frank R. Blattner

**Affiliations:** 1 Department of Plant and Animal Biology, Faculty of Biological Science and Technology, University of Isfahan, Isfahan 81746-73441, Iran; 2 Natural Resources Research Department, Isfahan Agricultural and Natural Resources Research and Education Center, AREEO, Isfahan 81785-199, Iran; a.akhavan@areeo.ac.ir; 3 Leibniz Institute of Plant Genetics and Crop Plant Research (IPK), 06466 Gatersleben, Germany; nemati@ipk-gatersleben.de (Z.N.); blattner@ipk-gatersleben.de (F.R.B.); 4 German Centre of Integrative Biodiversity Research (iDiv) Halle-Jena-Leipzig, 04103 Leipzig, Germany

**Keywords:** 2C value, *Astragalus* sect. *Hymenostegis*, chromosome number, flow cytometry, genome size, Leguminosae, nrDNA internal transcribed spacer region, polyploidy

## Abstract

*Astragalus* section *Hymenostegis* is one of the important characteristic elements of thorn-cushion formations in the Irano-Turanian floristic region. In this paper, we examined the chromosome number of 17 species (15 new reports) and provide estimates of genome size for 62 individuals belonging to 38 taxa of *A.* sect. *Hymenostegis*, some species outside this section, plus two *Oxytropis* species. Based on chromosome counts 11 species were found to be diploid (2*n* = 16), four species tetraploid (2*n* = 32) and two taxa hexaploid (2*n* = 48). From genome size measurements on silica-gel dried material, three ploidy levels (2*x*, 4*x* and 6*x*) were inferred, with a majority of species being diploid. The 2C values reach from 2.07 pg in diploid *Astragalus* *zohrabi* to 7.16 pg in hexaploid *A*. *rubrostriatus*. We found indications that species might occur with different cytotypes. A phylogenetic framework using nrDNA ITS sequences was constructed to understand the evolution of ploidy changes and genome sizes. It showed that genome size values among the studied taxa differ only slightly within ploidy levels and are nearly constant within most species and groups of closely related taxa within the genus *Astragalus*. The results of this study show that there is a rather strong correlation between genome sizes and chromosome numbers in sect. *Hymenostegis*. The resolution of the ITS-based phylogenetic tree is too low to infer evolutionary or environmental correlations of genome size differences. Polyploidization seems to contribute to the high species number in *Astragalus*, however, in sect. *Hymenostegis* it is not the main driver of speciation.

## 1. Introduction

Chromosomal characters play an important role in plant speciation [1,2]. Therefore, chromosome numbers and ploidy levels have been used to define evolutionary lineages and relationships between taxa [3,4,5]. Also genome-size changes often happen during speciation in plants [6,7,8,9] and might therefore provide insights in speciation processes. The genus *Astragalus* L. comprises about 2500–3000 species worldwide [10], with the highest number of endemic species occurring in Southwest Asia [11]. In Iran, the genus is represented by more than 850 species [10,11] distributed in different phytogeographical regions of the country. Relationships within *Astragalus* were studied for decades by molecular phylogenetic analyses [12,13,14,15,16,17,18] still some sectional and species-level relationships in *Astragalus* remain unanswered [14,15,16,17,18] and explanations for the high species number of the genus are lacking. *Astragalus* sect. *Hymenostegis* Bunge is one of the important spiny sections in the genus and was considered as an evolutionary young taxon undergoing a fast radiation [19]. With regard to the number of species, local endemism, and its distribution it seems that *A*. sect. *Hymenostegis* originated in Iran and diversified mainly in the northwest of the country [20,21].

Cytological work in several *Astragalus* taxa from all over the World have been published [22,23,24,25,26], however, detailed cytogenetic studies are scarce and analyses of chromosome numbers and karyotype structure together with genome size measurements are not yet available. According to previous studies, New World *Astragalus* species (Neo-Astragalus) have diverse basic chromosome numbers (*x* = 11, 12, 13, 14, 15) while in the Old World the majority of *Astragalus* species have *x* = 8, which is considered to be the ancestral basic number [27,28,29]. Based on IPCN [Index to Plant Chromosome Numbers, www.tropicos.org/Project/IPCN; accessed 8 December 2021], CCDB database [The Chromosome Counts Database, http://ccdb.tau.ac.il; accessed 8 December 2021] and Index to Plant Chromosome Numbers of Iran [30] chromosome numbers for the taxa in *A.* sect. *Hymenostegis* are unknown, except for a few species [31,32,33,34]. Taking into account the large number of *Astragalus* species and their worldwide distribution, genome size analyses are still rare [26,35,36,37,38]. According to the Plant DNA C-values Database at the Royal Botanic Gardens, Kew (https://cvalues.science.kew.org; accessed 22 November 2021), the genome sizes of only 39 taxa are available, which means that currently about 0.01% of the total number of species have been studied.

Because of the rarity of cytogenetic data and also the knowledge gap concerning genome size variation in *Astragalus* species we report here chromosome numbers and nuclear DNA content of *A.* sect. *Hymenostegis* species and some related taxa. To get insights on possible processes related to chromosome evolution, we put these data in a molecular phylogenetic framework for *A.* sect. *Hymenostegis*.

## 2. Results

### 2.1. Chromosome Numbers and Karyotypes

**Figure 1:**
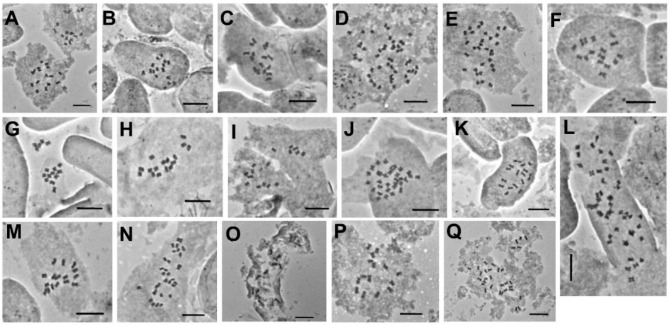
Mitotic metaphase chromosomes of *Astragalus* sect. *Hymenostegis*. (**A**) *A*. *assadabadensis* (2*n* = 2*x* = 16). (**B**) *A*. *chrysostachys* (2*n* = 2*x* = 16). (**C**) *A*. *hymenostegis* (2*n* = 2*x* = 16). (**D**) *A*. *lagopoides* (2*n* = 6*x* = 48). (**E**) *A*. *melanostictus* (2*n* = 4*x* = 32). (**F**) *A*. *nervistipulus* (2*n* = 2*x* = 16). (**G**) *A*. *paralurges* (2*n* = 2*x* = 16). (**H**) *A*. *pediculariformis* (2*n* = 2*x* = 16). (**I**) *A*. *pereshkhoranicus* (2*n* = 2*x* = 16). (**J**) *A*. *qorvehensis* (2*n* = 4*x* = 32). (**K**) *A*. *remotispicatus* (2*n* = 2*x* = 16). (**L**) *A*. *rubrostriatus* (2*n* = 6*x* = 48). (**M**) *A*. *seidabadensis* (2*n* = 2*x* = 16). (**N**) *A*. *sohrevardianus* (2*n* = 4*x* = 32). (**O**) *A*. *subrecognitus* (2*n* = 2*x* = 16). (**P**) *A. tabrizianus* (2*n* = 2*x* = 16). (**Q**) *A. tricholobus* (2*n* = 4*x* = 32). Scale bar = 10 µm.

**Figure 2:**
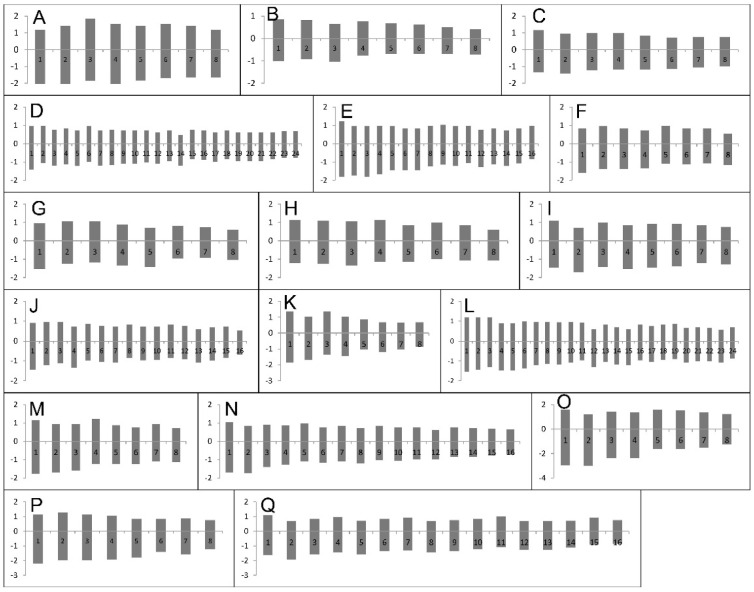
Idiograms of *Astragalus* sect. *Hymenostegis*. (**A**) *A*. *assadabadensis* (2*n* = 2*x* = 16). (**B**) *A*. *chrysostachys* (2*n* = 2*x* = 16). (**C**) *A*. *hymenostegis* (2*n* = 2*x* = 16). (**D**) *A*. *lagopoides* (2*n* = 6*x* = 48). (**E**) *A*. *melanostictus* (2*n* = 4*x* = 32). (**F**) *A*. *nervistipulus* (2*n* = 2*x* = 16). (**G**) *A*. *paralurges* (2*n* = 2*x* = 16). (**H**) *A*. *pediculariformis* (2*n* = 2*x* = 16). (**I**) *A*. *pereshkhoranicus* (2*n* = 2*x* = 16). (**J**) *A*. *qorvehensis* (2*n* = 4*x* = 32). (**K**) *A*. *remotispicatus* (2*n* = 2*x* = 16). (**L**) *A*. *rubrostriatus* (2*n* = 6*x* = 48). (**M**) *A*. *seidabadensis* (2*n* = 2*x* = 16). (**N**) *A*. *sohrevardianus* (2*n* = 4*x* = 32). (**O**) *A*. *subrecognitus* (2*n* = 2*x* = 16). (**P**) *A. tabrizianus* (2*n* = 2*x* = 16). (**Q**) *A. tricholobus* (2*n* = 4*x* = 32).

Chromosome numbers and karyotype characteristics for 17 *Astragalus* species belonging to sect. *Hymenostegis* are provided in Table 1, Figure 1 and Figure 2. This number is lower than the number of individuals we initially collected, as not in all cases (enough) seeds germinated to reliably infer karyotypes. Until now only for 12 taxa of section *Hymenostegis* chromosome numbers have been determined [31,32,33,34,39]. We here add new data for 15 species and confirm earlier chromosome counts for two species. The basic chromosome number in sect. *Hymenostegis* was established as *x* = 8 and the examined species belong to three ploidy levels: 11 taxa are diploid (2*n* = 2*x* = 16), four are tetraploid (2*n* = 4*x* = 32) and two were found to be hexaploid (2*n* = 6*x* = 48).

The total haploid chromosome lengths (CL) differed from 1.47 μm in *A. chrysostachys* to 3.5 μm in *A. subrecognitus*. The mean length for the long arms (P) of chromosomes varied from 0.81 μm in *A. chrysostachys* to 2.8 μm in *A. subrecognitus*. Mean lengths of short arms (q) ranged from 0.66 μm in *A*. *chrysostachys* to 1.44 μm in *A. assadabadensis* and the average values of chromosome arm ratios (r) ranged from 1.23 in *A*. *pediculariformis* to 1.77 in *A*. *tabrizianus*. TF% index has a perfect negative correlation with AsK%, and a perfect positive correlation with the Syi index. The Syi index has shown a perfect negative correlation with AsK%. *Astragalus chrysostachys* and *A*. *pediculariformis* have the highest values of TF% (45%) and *A*. *pediculariformis* has the highest Syi% (83%), as well as the lowest values of AsK% (54%), resulting in the most symmetric karyotype. The lowest value of TF% (36%) and Syi% (56%) and the highest value of AsK% (63%) belonged to *A*. *tabrizianus*. 

### 2.2. Genome Size Variation

Genome sizes for 62 individuals belonging to 38 *Astragalus* species and two *Oxytropis* taxa are provided here for the first time (Table 2). Coefficients of variation (CV) for the internal standard and sample peaks were in a range from 2.05% to 9.1% (mean = 3.37) and from 3.29% to 15.63% (mean = 7.79), respectively. Based on our results genome sizes vary from 1.54 pg/2C in *A. melanostictus* to 7.16 pg/2C in *A*. *rubrostriatus*. The 2C values of 60 individuals of 39 *Astragalus* species reported up to now in the Kew-based C-value database were in a range from 0.92 pg/2C in *A. epiglottis* to 13.25 pg/2C in *A. oleaefolius*.

### 2.3. Distribution of Genome Sizes among Phylogenetic Clades

**Figure 3:**
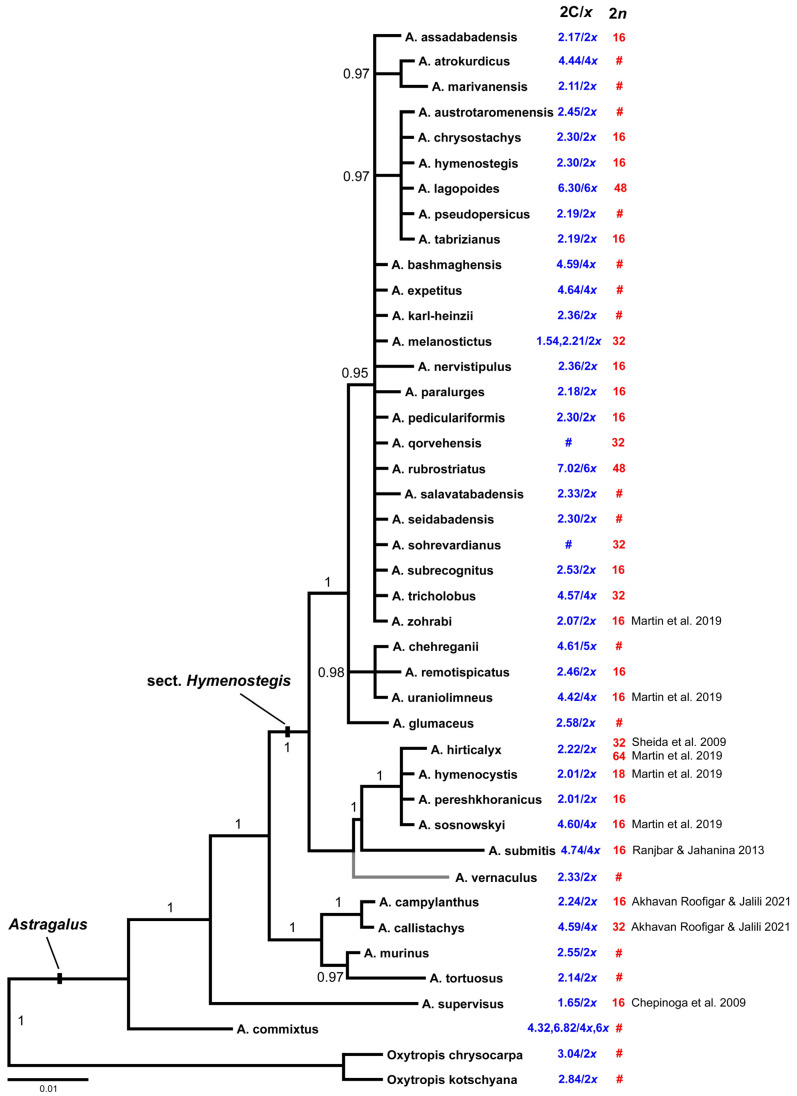
Phylogenetic tree resulting from Bayesian phylogenetic inference of nrDNA ITS sequences of *A*. sect. *Hymenostegis*. Numbers along branches provide Bayesian posterior probabilities. The numbers next to the taxon names show their averaged genome sizes in blue and chromosome numbers in red color. Values not available for certain taxa are depicted by #. *Astragalus vernaculus* was included in the tree according to Bagheri et al. [19]. Where no own chromosome counts were possible we added numbers taken from the literature [32,34,40,41,42].

To infer phylogenetic positions of the taxa with available chromosome number and/or genome size data, 40 *Astragalus* sequences of the nuclear ribosomal DNA internal transcribed spacer region (ITS, including the 5.8S rDNA region) were analyzed by maximum parsimony (MP) and Bayesian phylogenetic inference (BI). The sequences resulted in an alignment length of 607 base pairs (bp). Maximum parsimony analysis resulted in a single tree of 98 steps length (not shown) with a consistency index (CI) of 0.84 and a retention index (RI) of 0.91. Both MP and BI led to trees with matching topologies, so only the BI tree is provided in Figure 3. Resolution and support values for the closely related species of sect. *Hymenostegis* are generally low except for the two main clades of sect. *Hymenostegis,* the larger one comprising 28 species, the smaller one six. The taxonomically distant *Astragalus* taxa and two *Oxytropis* species were regarded as outgroups for the analysis.

In our own data we see a clear correlation of ploidy levels with genome sizes in 13 out of 14 cases where we have both values. Thus, diploids (2*n* = 2*x* = 16) have 2C genome sizes of 2.17 to 2.5 pg, tetraploids (2*n* = 4*x* = 32) have about twice as much nuclear DNA with 4.5 pg, and hexaploids (2*n* = 6*x* = 48) were found to have a 2C genome size of 6.8 to 7.2 pg. The only exception in our dataset is *A. melanostictus* where the individual used for chromosome counts had 32 chromosomes, while one of the two individuals used in flow cytometry presented 1.54 pg/2C, the smallest value in our analysis, and the second individual 2.21 pg/2C. For values taken from the literature we see major deviations from this otherwise consistent pattern (Figure 3), as in four out of nine cases we obtained 2C values indicating diploidy, while the reported chromosome numbers belong to tetra- and/or hexaploids or vice versa. There was no obvious pattern of genome size distribution across *Astragalus* groups and clades in section *Hymenostegis*.

## 3. Discussion

We established a comprehensive karyo-morphological analysis for *Astragalus* sect. *Hymenostegis* and provide for 39 *Astragalus* and two *Oxytropis* species genome-size values. All studied taxa, like most *Astragalus* species in the Old World, display a base chromosome number of *x* = 8. Three ploidy levels (2*x*, 4*x* and 6*x*) have been detected with 16, 32, and 48 chromosomes, respectively. Within our own analyzed species, we found a strong correlation between ploidy level and genome size. Thus, *Astragalus* species have on average a 2C of 2.28 (±0.17) pg for diploids, 4.56 (±0.12) pg for tetraploids and 6.79 (±0.31) pg for hexaploids. However, in *A. melanostictus* we found 2*n* = 4*x* = 32 chromosomes in the individual that was used for chromosome counts, while the two individuals used for genome-size estimations resulted (*i*) in highly disparate 2C values (1.54 vs. 2.21 pg) that (*ii*) fit only for the latter value to the genome-size classes defined above. As no leaf material was left for these individuals, we were not able to verify our measurements. We assume, however, that the smaller value might be due to a wrongly collected different taxon, as the result of our second individual (2.21 pg/2C) fits at least into the class of diploid genome sizes. Still, this doesn’t solve the disparity we see in this species regarding the 4x chromosome count with the otherwise diploid 2C value. Currently we assume that in *A. melanostictus* different cytotypes might exist and that we, by chance, used two different types for karyotype analysis and flow cytometry. The occurrence of different cytotypes within plant species is well known [43,44]. As we had to collect young leaves for genome-size determination early in the vegetation period (before onset of flowering) and the ripe seeds for chromosome counts when the remaining parts of the plants were nearly dry, it could happen that different plants were selected for leaf and seed collections. Similar differences we see quite often in the species where we took chromosome numbers from the literature (Figure 3) and measured genome size with leaves collected by us. Here too we have to assume either the occurrence of different cytotypes within the species, wrong species determinations, or that large and fast genome size changes happened and by chance the resulting 2C values fall anyway nicely within the ploidy-related size classes we could define by our own dataset. This latter assumption seems not very likely to us, while we cannot judge probabilities of the two former hypotheses.

Regarding relationships between different geographical areas or habitat types and the distribution of ploidy levels and/or of genome sizes, we found no clear trend. However, this could be caused by low phylogenetic resolution of the phylogeny and overall low range of variation of genome size among the analyzed species.

Although we had to rely on dried leaves for genome size measurements in *A.* sect. *Hymenostegis* taxa, the estimated 2C values where rather similar within ploidy levels and seem internally consistent even taking into account the relatively large CV values obtained for most of the individuals (Table 2). When excluding the deviating *A. melanostictus* measurement of 1.54 pg/2C, the genome-sizes differences within the studied *Astragalus* species are ≤5% where we measured multiple individuals, while differences among species within sect. *Hymenostegis* are in a range of 0–25% within the three (assumed) ploidy levels. While chromosome numbers are constant and genome sizes of *A.* sect. *Hymenostegis* did not show (larger) variation within the three ploidy levels, karyotype differences were more pronounced (Figure 2, Table 1). Still, as the karyotypes were mostly rather symmetrical, we cannot use them to discern if the polyploid taxa are of auto- or allopolyploid origin, which would be easier if the karyotypes or at least single chromosomes would be clearly different so that their occurrence could be traced back to certain diploid species and/or karyotypes. Similarly, also the ITS sequences are within sect. *Hymenostegis* species so similar that we cannot use them to identify parental progenitors [3] of the polyploid taxa. For this purpose, much faster evolving nuclear markers would be necessary. Still, as only about one third of the examined sect. *Hymenostegis* taxa are polyploids, polyploidy is contributing to the high speciation rates in this section [19] but seems not to be the main driver of the rapid and ongoing radiation of the group in the mountains of the Irano-Turanian floristic region. If our hypothesis should be true that within *Astragalus* species different ploidy levels exist, it would indicate even higher taxonomic diversity in the genus than already visible by the very large number of *Astragalus* species.

## 4. Materials and Methods

### 4.1. Plant Material

In total, for 42 species (66 individuals) leaves and/or seeds were collected from natural habitats throughout 2017 to 2019. To obtain young leaves and ripe seeds collections had to be done at least at two different time points in the vegetation period. Details concerning the examined taxa are accessible in Table 2. Voucher specimens of these species were deposited in the herbarium of the University of Isfahan (HUI).

### 4.2. Chromosome Number and Karyotype Analysis

Seeds of the plants were placed on filter paper in petri dishes and kept wet till germination. Root tips of up to ten germinated seeds per individual of about 0.5–1 cm in length were pretreated with 0.01% aqueous solution of colchicine for 3–4 h at 4 °C, and fixed in freshly prepared absolute ethanol-acetic acid (*v*/*v*, 3:1) solution. Fixed root tips were washed three times in distilled water and kept at 4 °C in 70% ethanol for several months, until use. Then, meristems were hydrolyzed in 1N HCl at 60 °C for 14 min, stained, and squashed in a drop of aceto-orcein as follow. Fixed root tips were kept in 2% aceto-orcein for 3 h and incubated for 10–15 min in cellulase-pectinase enzyme solution at 37 °C. The stained roots were squashed in 45% acetic acid under a stereomicroscope and the best metaphase plates were photographed in an Olympus (BX40) microscope. Chromosome counts were made on well-spread metaphase plates. The karyotype was prepared according to metaphase plates. Designation of centromere position and chromosome type was done according to Levan et al. [45] and Stebbins [46]. The mean total length (TL) of chromosomes, the arms ratio (r = long arm/short arm), the centromeric index [Ci = 100 × short arm/(long + short arm)], asymmetry index [Ai% = (Σ long arms/Σ total chromosome lengths) × 100, according to Arano and Saito [47], and symmetry class according to Stebbins [46] were evaluated. Overall, the parameters were very similar within species and we found no significant variation among them, so we used the best metaphase to calculate the final karyotype parameters.

### 4.3. Genome Size Measurement

Genome size was determined using propidium iodide (PI) flow cytometry with a Cyflow Space (Partec GmbH, Münster, Germany), following the procedure described in Jakob et al. [8]. We used *Zea mays* (5.43 pg/2C) as standard [48] and the buffer CyStain PI Absolute P (Partec GmbH, Münster, Germany). Depending on the available materials between one and four individuals were measured for each taxon (Table 2). As the *A.* sect. *Hymenostegis* species are generally hard to cultivate and occur naturally at remote locations we used dried leaf material for genome size measurements. Silica-gel dried material has previously been shown to be suitable for measuring genome size in some cases [49], although the lower quality of data obtained with this material has often limited its use to inferring ploidy level [50]. For some *Astragalus* species, we measured both silica-gel dried and fresh leaves to evaluated the impact of material type on flow cytometry measurements (data not shown). We found semiarid- to arid-adapted species (i.e., the majority of spiny taxa) generally suitable for flow cytometry with silica-gel preserved material if leaves were collected at a young stage, dried fast and measured shortly afterwards or stored frozen at −20 °C. Given the CVs in the flow histogram peaks were often >5% (Table 2) and we did not perform statistical tests to compare the values obtained with fresh vs. silica gel preserved material, further studies based on fresh plants and with a larger sampling would be interesting in order to confirm the current results.

### 4.4. Phylogenetic Analyses

Molecular phylogenetic analyses of *A*. sect. *Hymenostegis* were based on our previous dataset [19], which used sequences of the nuclear rDNA internal transcribed spacer (ITS) region to infer phylogenetic relationships. In total for 40 *Astragalus* species where we had chromosome counts and/or 2C genome-size values, ITS sequences of *Astragalus* (33 species from sect. *Hymenostegis* and seven species from other sections) plus two *Oxytropis* species as outgroups, were aligned and subjected to phylogenetic analyses. Bayesian phylogenetic inference (BI) was done in MrBayes 3.1 [51]. The sequence evolution model (SYM + G) was chosen following the Akaike Information Criterion (AIC) inferred in PAUP* 4.0a152 [52]. For BI two times four Markov Chain Monte Carlo (MCMC) analyses was run for 4 million generations, with a sampling of trees every 1000 generations. The first 25% of trees were discarded as burn-in. The remaining trees were summarized with MrBayes. Finally, the phylogenetic trees were visualized using FigTree 1.3.1 (http://tree.bio.ed. ac.uk; accessed 18 October 2021). The MP analysis was conducted in PAUP* using the heuristic search algorithm. To test clade support, a bootstrap analysis with 500 bootstrap resamples was conducted. A list of examined taxa and GenBank accession numbers of the ITS sequences are listed in Table 2.

## 5. Conclusions

The current work presents the first set of genome size and chromosomal data for *A.* sect. *Hymenostegis*. We inferred three ploidy levels (2*x*, 4*x*, 6*x*) with the majority of analyzed taxa being diploids. Genome sizes were quite similar within the di- and tetraploids, while larger variation was found in hexaploids. As sect. *Hymenostegis* species are quite young, the phylogenetic resolution of the ITS dataset is low. This, together with the relative uniformity of 2C values, prevents assigning genome sizes differences to specific clades in the section or to relate them to environmental parameters. Polyploidization is involved but seems not the major driver of species diversification in sect. *Hymenostegis*, as about one third of the analyzed taxa are polyploids.

## Figures and Tables

**Table 1:** Chromosome counts and karyo-morphological parameters of the examined *Astragalus* taxa.

No.	Species	Ind./plates	Chr. No.	CL ^1^	q ^2^	P ^3^	TF% ^4^	S% ^5^	Ask% ^6^	Syi% ^7^	R ^8^	Ci ^9^	KF ^10^
1	*A*. *assadabadensis*	7/11	2*n* = 2*x* = 16	3.4	1.44	1.95	42	74	57	74	1.38	0.42	1M + 6m + 1sm
2	*A*. *chrysostachys*	3/5	2*n* = 2*x* = 16	1.47	0.66	0.81	45	61	55	81	1.26	0.43	2M + 5m + 1sm
3	*A*. *hymenostegis*	10/14	2*n* = 2*x* = 16	2.08	0.89	1.19	43	69	57	75	1.34	0.42	8m
4	*A*. *lagopoides*	5/9	2*n* = 6*x* = 48	1.74	0.72	1.02	41	61	58	70	1.44	0.41	1M + 21m + 2sm
5	*A*. *melanostictus*	9/8	2*n* = 4*x* = 32	2.27	0.93	1.34	41	60	59	69	1.44	0.42	13m + 3sm
6	*A*. *nervistipulus*	6/8	2*n* = 2*x* = 16	2.09	0.82	1.26	39	69	60	65	1.57	0.41	5m + 3sm
7	*A*. *paralurges*	9/11	2*n* = 2*x* = 16	2.06	0.86	1.2	41	66	58	71	1.43	0.39	6m + 2sm
8	*A*. *pediculariformis*	6/11	2*n* = 2*x* = 16	2.12	0.96	1.15	45	71	54	83	1.23	0.45	2M + 5m + 1sm
9	*A*. *pereshkhoranicus*	6/9	2*n* = 2*x* = 16	2.32	0.88	1.43	38	80	62	61	1.65	0.41	6m + 2sm
10	*A*. *qorvehensis*	8/9	2*n* = 4*x* = 32	1.79	0.77	1.02	43	52	57	75	1.34	0.41	2M + 12m + 2sm
11	*A*. *remotispicatus*	10/8	2*n* = 2*x* = 16	2.26	0.95	1.3	42	47	58	73	1.39	0.43	1M + 6m + 1sm
12	*A*. *rubrostriatus*	5/9	2*n* = 6*x* = 48	2.01	0.85	1.16	42	57	57	73	1.4	0.44	2M + 18m + 4sm
13	*A*. *seidabadensis*	10/8	2*n* = 2*x* = 16	2.31	0.94	1.36	41	63	59	69	1.46	0.38	1M + 5m + 2sm
14	*A*. *sohrevardianus*	7/10	2*n* = 4*x* = 32	1.91	0.79	1.12	41	50	58	71	1.40	0.36	15m + 1sm
15	*A*. *subrecognitus*	10/7	2*n* = 2*x* = 16	3.5	1.41	2.8	40	53	59	68	1.48	0.38	2M + 3m + 3sm
16	*A*. *tabrizianus*	5/7	2*n* = 2*x* = 16	2.75	0.99	1.76	36	59	63	56	1.77	0.38	3m + 5sm
17	*A*. *tricholobus*	8/7	2*n* = 4*x* = 32	2.16	0.82	1.33	38	58	62	61	1.66	0.43	1M + 8m + 7sm

^1^ CL: chromosome length; ^2^ q: short arm; ^3^ P: long arm; ^4^ TF%: total form percentage; ^5^ S%: symmetry index; ^6^ AsK%: Arano index of karyotype asymmetry; ^7^ Syi%: index of karyotype symmetry; ^8^ R: the arms ratio; ^9^ Ci: the centromeric index; ^10^ KF: Karyotype Formulae.

**Table 2:** List of examined taxa and voucher specimens with 2C DNA values in picogram, and the related genome-size measurement parameters used in this study. Specimens used for chromosome counting were marked by asterisks. Accession numbers of ITS sequences refer to the GenBank nucleotide database.

Species	Section	Locality	Geographical Coordinates	Elevation	Genome Size Standard	Mean Standard	Mean Sample	CV % Standard	CV % Sample	2C Genome Size (pg)	GenBank Acc. No. ITS
*A. assadabadensis* F.Ghahrem. & Podlech *	*Hymenostegis*	Hamadan, Assadabad	34°49′70″ N 48°10′90″ E	2270 m	Maize	103.79	41.55	2.89	7.39	2.17	LT622371
*A. atrokurdicus* Maassoumi, F.Ghahrem., Bagheri & Podlech	*Hymenostegis*	Zanjan, Zarand	36°09′18″ N 48°30′78″ E	2040 m	Maize	100.46	82.66	4.11	3.93	4.47	LT622374
*A. austrotaromensis* Maassoumi, F.Ghahrem., Bagheri & Podlech *1*	*Hymenostegis*	Zanjan, Tarom	36°45′74″ N 48°49′90″ E	2150 m	Maize	97.78	44.89	5.13	13.66	2.49	LT622379
*A. austrotaromensis* Maassoumi, F.Ghahrem., Bagheri & Podlech *2*	*Hymenostegis*	Zanjan, Tarom	36°45′88″ N 48°48′86″ E	2120 m	Maize	98.71	44.07	6	9.29	2.42	-
*A. bashmaghensis* Maassoumi & Podlech *1*	*Hymenostegis*	Kordestan, Marivan to Saqez	36°12′08″ N 46°31′35″ E	1800 m	Maize	99.82	82.07	2.18	5.8	4.46	LT622380
*A. bashmaghensis* Maassoumi & Podlech *2*	*Hymenostegis*	Kordestan, Marivan to Saqez	36°12′08″ N 46°31′35″ E	1810 m	Maize	99.76	86.54	2.32	6.3	4.71	-
*A. callistachys* Buhse	*Microphysa*	Isfahan, Meymeh	33°36′76″ N 50°59′79″ E	2110 m	Maize	105.69	89.41	2.86	4.22	4.59	LT622397
*A. campylanthus* Boiss.	*Campylanthus*	Isfahan, Boien	33°10′65″ N 50°17′06″ E	2835 m	Maize	107.48	44.4	4.62	15.56	2.24	LT622398
*A. chehreganii* Zarre & Podlech	*Hymenostegis*	West Azerbaijan, Qoshchi	38°02′14″ N 44°57′84″ E	1660 m	Maize	100.12	85	2.86	4.57	4.61	LT622401
*A. chrysostachys* Boiss. *1* *	*Hymenostegis*	Zanjan, Mahneshan	36°49′86″ N 47°25′36″ E	2090 m	Maize	96.88	40.73	2.12	5.38	2.28	LT622405
*A. chrysostachys* Boiss. *2*	*Hymenostegis*	Zanjan, Mahneshan	36°49′86″ N 47°25′36″ E	2095 m	Maize	95.07	39.75	2.25	3.64	2.27	-
*A. chrysostachys* Boiss. *3*	*Hymenostegis*	East Azerbaijan, Saeidabad	37°54′22″ N 46°32′01″ E	2010 m	Maize	102.08	42.77	2.63	4.62	2.28	-
*A. chrysostachys* Boiss. *4*	*Hymenostegis*	Zanjan, Mahneshan	36°49′86″ N 47°25′36″ E	2085 m	Maize	92.23	40.35	9.1	6.22	2.38	-
*A. commixtus* Bunge *1*	*Ankylotus*	Zanjan, Abhar	36°07′01″ N 49°03′56″ E	1910 m	Maize	71.75	90.1	5.66	6.54	6.82	AB051925
*A. commixtus* Bunge *2*	*Ankylotus*	Zanjan, Abhar	36°07′01″ N 49°03′56″ E	1912 m	Maize	90.1	71.75	6.54	5.66	4.32	-
*A. expetitus* Maassoumi	*Hymenostegis*	Zanjan, Anguran	36°36′16″ N 47°20′07″ E	2460 m	Maize	107.56	91.85	2.59	4.58	4.64	LT622430
*A. glumaceus* Boiss. *1*	*Hymenostegis*	Zanjan, Mahneshan	36°51′43″ N 47°26′17″ E	1910 m	Maize	94.97	45.5	3.2	7.18	2.60	LT622439
*A. glumaceus* Boiss. *2*	*Hymenostegis*	Kordestan, Sonateh	36°10′60″ N 46°33′71″ E	1510 m	Maize	92.6	43.69	8.5	9.32	2.56	-
*A. hirticalyx* Boiss. & Kotschy *1*	*Hymenostegis*	West Azerbaijan, Khoy to Chaldoran	38°40′45″ N 44°41′99″ E	1915 m	Maize	98.46	41.49	3.01	14	2.29	LT622447
*A. hirticalyx* Boiss. & Kotschy *2*	*Hymenostegis*	West Azerbaijan, Chaldoran	38°40′45″ N 44°41′99″ E	1905 m	Maize	99.01	39.38	4.06	10.32	2.16	-
*A. hymenocystis* Fisch. & C.A.Mey.	*Hymenostegis*	West Azerbaijan, Qoshchi	38°02′14″ N 44°57′84″ E	1660 m	Maize	98.2	36.28	4.37	9.4	2.01	LT622454
*A. hymenostegis* Fisch. & C.A.Mey. 1*	*Hymenostegis*	West Azerbaijan, Hashtian to Salmas	38°01′39″ N 44°47′67″ E	1800 m	Maize	97.38	39.64	2.81	8.86	2.21	LT622455
*A. hymenostegis* Fisch. & C.A.Mey. *2*	*Hymenostegis*	West Azerbaijan, Qoshchi	38°02′14″ N 44°57′84″ E	1670 m	Maize	100.09	43.8	2.91	7.73	2.38	-
*A. karl-heinzii* Maassoumi *1*	*Hymenostegis*	Zanjan, Zarand	36°09′07″ N 48°30′88″ E	2090 m	Maize	98.62	42.52	2.63	5.39	2.34	KT997422
*A. karl-heinzii* Maassoumi *2*	*Hymenostegis*	Zanjan, Zarand	36°09′07″ N 48°30′88″ E	2092 m	Maize	98.22	42.92	3.09	12.49	2.37	-
*A. lagopoides* Lam. *	*Hymenostegis*	East Azerbaijan, Kuhkamar	38°37′22″ N 45°53′37″ E	2080 m	Maize	101.34	117.52	2.9	4.73	6.30	LT622474
*A. marivanensis* Podlech & Maassoumi	*Hymenostegis*	Kordestan, Marivan to Saqez	36°09′44″ N 46°19′30″ E	1730 m	Maize	95.93	37.21	2.71	5.35	2.11	LT622496
*A. melanostictus* Freyn *1* *	*Hymenostegis*	Zanjan, Mahneshan	36°39′44″ N 47°36′12″ E	2160 m	Maize	96.98	27.46	2.31	6.23	1.54	LT622503
*A. melanostictus* Freyn *2*	*Hymenostegis*	Zanjan, Dandi to Tekab	36°35′26″ N 47°31′28″ E	1960 m	Maize	94.33	38.41	2.74	6.74	2.21	-
*A. murinus* Boiss.	*Anthylloidei*	Isfahan, Boien	33°10′65″ N 50°17′06″ E	2800 m	Maize	109.86	51.54	4.48	8.13	2.55	LT622519
*A. nervistipulus* Boiss. & Hausskn. *	*Hymenostegis*	Kordestan, Sanandaj to Marivan	35°25′03″ N 46°51′34″ E	2040 m	Maize	101.86	44.28	4.09	9.96	2.36	LT622524
*A. paralurges* Bunge *1* *	*Hymenostegis*	Zanjan, Qeydar	36°07′19″ N 48°32′48″ E	2400 m	Maize	101.33	40.97	2.89	7.52	2.20	LT622534
*A. paralurges* Bunge *2*	*Hymenostegis*	Zanjan, Zarand	36°09′07″ N 48°30′88″ E	2090 m	Maize	103	41.03	2.93	8.64	2.16	-
*A. pediculariformis* Maassoumi *1* *	*Hymenostegis*	Zanjan, Qeydar	36°07′19″ N 48°32′48″ E	2402 m	Maize	99.17	42.6	2.19	15.63	2.33	LT622543
*A. pediculariformis* Maassoumi *2*	*Hymenostegis*	Zanjan, Qeydar	36°07′19″ N 48°32′48″ E	2405 m	Maize	101.82	42.4	2.41	5.63	2.26	-
*A. pereshkhoranicus* Maassoumi & F.Ghahrem. *	*Hymenostegis*	West Azerbaijan, Mavana to Serow	37°36′74″ N 44°47′66″ E	1500 m	Maize	99	36.63	3.37	13.81	2.01	LT622550
*A. pseudopersicus* Podlech & Maassoumi *1*	*Hymenostegis*	East Azerbaijan, Sofian	38°15′78″ N 45°51′11″ E	1545 m	Maize	108.18	43.46	2.35	15.41	2.18	LT622560
*A. pseudopersicus* Podlech & Maassoumi *2*	*Hymenostegis*	East Azerbaijan, Sofian	38°15′78″ N 45°51′11″ E	1550 m	Maize	108.56	43.93	2.54	6.95	2.20	-
*A*. *qorvehensis* Podlech *	*Hymenostegis*	Hamadan, Qorveh	34°49′70″ N 48°10′90″ E	2280 m	-	-	-	-	-	-	LT622565
*A. remotispicatus* Bagheri & Maassoumi *	*Hymenostegis*	Zanjan, Zarand	36°09′07″ N 48°30′88″ E	2085 m	Maize	101.27	45.85	2.51	10.75	2.46	KT997427
*A. rubrostriatus* Bunge *1* *	*Hymenostegis*	Zanjan, Tarom	36°45′74″ N 48°49′90″ E	2140 m	Maize	104.93	132.77	4.12	5.63	6.87	LT622579
*A. rubrostriatus* Bunge *2*	*Hymenostegis*	Zanjan, Mahneshan	36°49′86″ N 47°25′36″ E	2090 m	Maize	102.25	134.82	5.92	6.4	7.16	-
*A. salavatabadensis* Podlech *1*	*Hymenostegis*	Kordestan, Salavatabad	35°16′50″ N 47°08′40″ E	2030 m	Maize	97.82	42.51	2.58	14.68	2.36	LT622589
*A. salavatabadensis* Podlech *2*	*Hymenostegis*	Kordestan, Salavatabad	35°16′50″ N 47°08′40″ E	2040 m	Maize	98.35	41.68	2.67	8.35	2.30	-
*A. seidabadensis* Bunge *1* *	*Hymenostegis*	East Azerbaijan, Kandowan	37°53′01″ N 46°11′22″ E	1750 m	Maize	107.19	45.89	3.17	10.13	2.32	LT622595
*A. seidabadensis* Bunge *2*	*Hymenostegis*	East Azerbaijan, Kandowan	37°53′01″ N 46°11′22″ E	1758 m	Maize	100.86	43.84	3.2	12.04	2.36	-
*A. seidabadensis* Bunge *3*	*Hymenostegis*	East Azerbaijan, Kandowan	37°53′01″ N 46°11′22″ E	1755 m	Maize	99.1	40.61	6.14	7.77	2.23	-
*A. sohrevardianus* Bagheri, Maassoumi & F.Ghahrem. *	*Hymenostegis*	Zanjan, Sohrevard	36°05′33″ N 48°28′78″ E	2020 m	-	-	-	-	-	-	LT622598
*A. sosnowskyi* Grossh. *1*	*Hymenostegis*	Kordestan, Bukan to Mahabad	36°12′50″ N 46°31′40″ E	1800 m	Maize	101.12	87.27	2.45	5.66	4.69	LT622602
*A. sosnowskyi* Grossh. *2*	*Hymenostegis*	Kordestan, Bukan to Mahabad	36°12′50″ N 46°31′40″ E	1810 m	Maize	97.17	80.67	2.96	4.77	4.51	-
*A. submitis* Boiss.	*Hymenostegis*/*Anthylloidei*	Zanjan, Zarand	36°09′07″ N 48°30′88″ E	2090 m	Maize	96.81	84.59	2.17	3.91	4.74	LT622611
*A. subrecognitus* Bagheri, Maassoumi & F.Ghahrem. *1* *	*Hymenostegis*	Zanjan, Mahneshan to Pari	36°51′43″ N 47°26′17″ E	1900 m	Maize	103.36	49.07	2.36	7.48	2.58	-
*A. subrecognitus* Bagheri, Maassoumi & F.Ghahrem. *2*	*Hymenostegis*	Zanjan, Mahneshan to Pari	36°51′43″ N 47°26′17″ E	1905 m	Maize	100.5	45.96	2.54	7.77	2.48	LT622615
*A. supervisus* (Kuntze) E.Sheld.	*Incani*	Zanjan, Zarand	36°09′07″ N 48°30′88″ E	2080 m	Maize	96.85	29.51	3.05	5.71	1.65	AB231116
*A. tabrizianus* Fisch. *	*Hymenostegis*	Kordestan, Bukan to Mahabad	36°45′22″ N 45°52′40″ E	1930 m	Maize	99.65	40.18	3.5	11.97	2.19	LT622619
*A. tortuosus* DC.	*Anthylloidei*	Kordestan, Sanandaj to Marivan	35°25′03″ N 46°51′34″ E	2045 m	Maize	100.76	39.76	2.72	5.77	2.14	AB908451
*A. tricholobus* DC. *1* *	*Hymenostegis*	Zanjan, Qeydar	36°07′19″ N 48°32′48″ E	2400m	Maize	100.12	83.07	2.05	3.69	4.51	LT622623
*A. tricholobus* DC. *2*	*Hymenostegis*	Zanjan, Mahneshan	36°49′86″ N 47°25′36″ E	2085 m	Maize	106.19	91.9	2.18	6.95	4.70	-
*A. tricholobus* DC. *3*	*Hymenostegis*	Zanjan, Abhar	36°07′01″ N 49°03′56″ E	1910 m	Maize	99.62	81.91	2.31	5.73	4.46	-
*A. tricholobus* DC. *4*	*Hymenostegis*	Zanjan, Mahneshan to Pari	36°51′43″ N 47°26′17″ E	1900 m	Maize	105.11	89.07	2.48	4.3	4.60	-
*A. uraniolimneus* Boiss.	*Hymenostegis*	East Azerbaijan, Kuhkamar	38°38′58″ N 45°55′14″ E	2280 m	Maize	97.09	79.09	2.42	3.29	4.42	LT622627
*A. vernaculus* Podlech *1*	*Hymenostegis*	Isfahan, Boien	33°10′65″ N 50°17′04″ E	2800 m	Maize	103.65	43.36	2.84	13.59	2.27	LT622633
*A. vernaculus* Podlech *2*	*Hymenostegis*	Isfahan, Dehrajab	33°09′30″ N 50°16′91″ E	2730 m	Maize	96.61	42.38	3.09	6.58	2.38	-
*A. zohrabi* Bunge	*Hymenostegis*	West Azerbaijan, Hashtian to Salmas	38°01′39″ N 44°47′67″ E	1805 m	Maize	100.14	38.14	3.4	7.73	2.07	LT622637
*Oxytropis chrysocarpa* Boiss.	-	Isfahan, Fereydunshahr	32°56′14″ N 50°02′94″ E	2920 m	Maize	106.11	59.34	3.1	5.99	3.04	LC213337
*Oxytropis kotschyana* Boiss. & Hohen.	-	Zanjan, Qeydar	36°07′19″ N 48°32′48″ E	2405 m	Maize	105.35	55.09	3.8	5.49	2.84	LT622640

## Data Availability

The data presented in this study are available in the article.

## References

[1] Lysak M.A., Berr A., Pecinka A., Schmidt R., McBreen K., Schubert I. (2006). Mechanisms of chromosome number reduction in *Arabidopsis thaliana* and related Brassicaceae species. Proc. Natl. Acad. Sci. USA.

[2] Weiss-Schneeweiss H., Schneeweiss G.M. (2013). Karyotype diversity and evolutionary trends in angiosperms. Plant Genome Diversity 2: Physical Structure, Behaviour and Evolution of Plant Genomes.

[3] Blattner F.R. (2004). Phylogeny of *Hordeum* (Poaceae) as inferred by nuclear rDNA ITS sequences. Mol. Phylogenet. Evol..

[4] Bennett M.D., Leitch I.J. (2011). Nuclear DNA amounts in angiosperms: Targets, trends and tomorrow. Ann. Bot..

[5] Jang T.S., Parker J.S., Emadzade K., Temsch E.M., Leitch A.R., Weiss-Schneeweiss H. (2018). Multiple origins and nested cycles of hybridization result in high tetraploid diversity in the monocot *Prospero*. Front. Plant Sci..

[6] Greilhuber J. (1998). Intraspecific variation in genome size: A critical reassessment. Ann. Bot..

[7] Soltis D.E., Soltis P.S., Bennett M.D., Leitch I.J. (2003). Evolution of genome size in the angiosperms. Am. J. Bot..

[8] Jakob S.S., Meister A., Blattner F.R. (2004). The considerable genome size variation in *Hordeum* species (Poaceae) is linked to phylogeny, life form, ecology, and speciation rates. Mol. Biol. Evol..

[9] Kellogg E.A., Bennetzen J.L. (2004). The evolution of nuclear genome structure in seed plants. Am. J. Bot..

[10] Podlech D., Zarre S. (2013). A Taxonomic Revision of the Genus Astragalus L. (Leguminosae) in the Old World.

[11] Maassoumi A.A. (1998). Old World Check-List of Astragalus.

[12] Sanderson M.J., Wojciechowski M.F. (2000). Improved bootstrap confidence limits in large-scale phylogenies, with an example from Neo-Astragalus (Leguminosae). Syst. Biol..

[13] Wojciechowski M.F. (2005). *Astragalus* (Fabaceae): A molecular phylogenetic perspective. Brittonia.

[14] Kazempour Osaloo S., Maassoumi A.A., Murakami N. (2003). Molecular systematic of the genus *Astragalus* L. (Fabaceae): Phylogenetic analyses of nuclear ribosomal DNA internal transcribed spacers and chloroplast gene *ndh*F sequences. Plant Syst. Evol..

[15] Kazempour Osaloo S., Maassoumi A.A., Murakami N. (2005). Molecular systematics of the Old World *Astragalus* (Fabaceae) as inferred from nrDNA ITS sequence data. Brittonia.

[16] Azani N., Bruneau A., Wojciechowski M.F., Zarre S. (2017). Molecular phylogenetics of annual *Astragalus* (Fabaceae) and its systematic implications. Bot. J. Linn. Soc..

[17] Azani N., Bruneau A., Wojciechowski M.F., Zarre S. (2019). Miocene climate change as a driving force for multiple origins of annual species in *Astragalus* (Fabaceae, Papilionoideae). Mol. Phylogenet. Evol..

[18] Su C., Duan L., Liu P., Liu J., Chang Z., Wen J. (2021). Chloroplast phylogenomics and character evolution of eastern Asian *Astragalus* (Leguminosae): Tackling the phylogenetic structure of the largest genus of flowering plants in Asia. Mol. Phylogenet. Evol..

[19] Bagheri A., Maassoumi A.A., Rahiminejad M.R., Brassac J., Blattner F.R. (2017). Molecular phylogeny and divergence times of *Astragalus* section *Hymenostegis*: An analysis of a rapidly diversifying species group in Fabaceae. Sci. Rep..

[20] Bagheri A., Rahiminejad M.R., Maassoumi A.A. (2014). A new species of the genus *Astragalus* (Leguminosae-Papilionoideae) from Iran. Phytotaxa.

[21] Bagheri A., Maassoumi A.A., Rahiminejad M.R., Blattner F.R. (2016). Molecular phylogeny and morphological analysis support a new species and new synonymy in Iranian *Astragalus* (Leguminosae). PLoS ONE.

[22] Manandhar L., Sakya S.R. (2004). Cytotaxonomic studies in two species of *Astragalus*. J. Cytol. Genet..

[23] Badr A., Sharawy S.M. (2007). Karyotype analysis and systematic relationships in the Egyptian *Astragalus* L. (Fabaceae). Int. J. Bot..

[24] Martin E., Duran A., Dinç M., Erişen S., Babaoğlu M. (2008). Karyotype analyses of four *Astragalus* L. (Fabaceae) species from Turkey. Phytologia.

[25] Yousefzadeh K., Houshmand S., Zamani Dadane G. (2010). Karyotype analysis of *Astragalus effusus* Bunge (Fabaceae). Caryologia.

[26] Abdel Samad F., Baumel A., Juin M., Pavon D., Siljak-Yakovlev S., Médail F., Bou Dagher Kharrat M. (2014). Phylogenetic diversity and genome sizes of *Astragalus* (Fabaceae) in the Lebanon biogeographical crossroad. Plant Syst. Evol..

[27] Ledingham G.F., Fahselt M.D. (1964). Chromosome numbers of some North American species of *Astragalus* (Leguminosae). Sida.

[28] Ledingham G.F., Rever B.M. (1963). Chromosome numbers of some Southwest Asian species of *Astragalus* and *Oxytropis* (Leguminosae). Canad. J. Genet. Cytol..

[29] Wojciechowski M.F., Sanderson M.J., Hu J.M. (1999). Evidence on the monophyly of *Astragalus* (Fabaceae) and its major subgroups based on nuclear ribosomal DNA ITS and chloroplast DNA *trn*L intron data. Syst. Bot..

[30] Ghaffari S.M. (2020). Index to Plant Chromosome Number of Iran.

[31] Sheidai M., Gharemaninejad F. (2008). New chromosome number and karyotype analysis in four *Astragalus* L. (Fabaceae) species. Iran. J. Bot..

[32] Sheidai M., Zarre S., Ismeilzadeh J. (2009). New chromosome number reports in tragacanthic *Astragalus* species. Caryologia.

[33] Bagheri A., Erkul S.K., Maassoumi A.A., Rahiminejad M.R., Blattner F.R. (2015). *Astragalus trifoliastrum* (Fabaceae), a revived species for the flora of Turkey. Nord. J. Bot..

[34] Martin E., Icyer Dogan G., Karaman Erkul S., Eroglu H.E. (2019). Karyotype analyses of 25 Turkish taxa of *Astragalus* from the sections *Macrophyllum*, *Hymenostegis*, *Hymenocoleus*, and *Anthyllis* (Fabaceae). Turk. J. Bot..

[35] Siljak-Yakovlev S., Pustahija F., Solic E.M., Bogunić F., Muratović E., Bašić N., Brown S.C. (2010). Towards a genome size and chromosome number database of Balkan flora: C-values in 343 taxa with novel values for 242. Adv. Sci. Lett..

[36] Temsch E.M., Temsch W., Ehrendorfer-Schratt L., Greilhuber J. (2010). Heavy metal pollution, selection, and genome size: The species of the Žerjav Study revisited with flow cytometry. J. Bot..

[37] Bou Dagher-Kharrat M., Siljak-Yakovlev S., Abdel-Samad N., Douaihy B.C., Abdel-Samad F., Bourge M., Brown S.C. (2013). Nuclear DNA C-values for biodiversity screening: Case of the Lebanese flora. Plant Biosyst..

[38] Vallès J., Bašić N., Bogunić F., Bourge M., Brown S.C., Garnatje T., Hajrudinović A., Muratović E., Pustahija F., Šolić E.M. (2014). Contribution to plant genome size knowledge: First assessments in five genera and 30 species of angiosperms from western Balkans. Bot. Serb..

[39] Ranjbar M., Assadi A., Karamian R. (2011). Systematic study of *Astragalus chrysostachys* Boiss. (Fabaceae) in Iran, with the description of a new species. Ann. Nat. Hist. Mus. Wien Ser. B Bot. Zool..

[40] Ranjbar M., Jahanian S. (2013). Cytotaxonomic study of *Astragalus* sect. Megalocystis (Fabaceae) in Iran. Cytologia.

[41] Akhavan Roofigar A., Jalili A. (2021). A new chromosome number report in five endemic *Astragalus* L. (Fabaceae) species of Iran. Iran. J. Bot..

[42] Chepinoga V.V., Aleksandr A.G., Enushchenko I.V., Rosbakh S.A. (2009). IAPT/IOPB chromosome data 8. Taxon.

[43] Brassac J., Blattner F.R. (2015). Species-level phylogeny and polyploid relationships in *Hordeum* (Poaceae) inferred by next-generation sequencing and in silico cloning of multiple nuclear loci. Syst. Biol..

[44] Soltis D.E., Visger C.J., Marchant D.B., Soltis P.S. (2016). Polyploidy: Pitfalls and paths to a paradigm. Am. J. Bot..

[45] Levan A., Fredgra K., Sandberg A.A. (1964). Nomenclature for centromeric position on chromosomes. Hereditas.

[46] Stebbins G.L. (1971). Chromosomal Evolution in Higher Plants.

[47] Arano H., Saito H. (1980). Cytological studies in family Umbelliferae 5. Karyotypes of seven species in subtribe Seselinae. La Kromosomo.

[48] Lysák M.A., Doležel J. (1998). Estimation of nuclear DNA content in *Sesleria* (Poaceae). Caryologia.

[49] Farhat P., Hidalgo O., Robert T., Siljak-Yakovlev S., Leitch I.J., Adams R.P., Bou Dagher-Kharrat M. (2019). Polyploidy in the conifer genus *Juniperus*: An unexpectedly high rate. Front. Plant Sci..

[50] Farhat P., Siljak-Yakovlev S., Hidalgo O., Rushforth K., Bartel J.A., Valentin N., Leitch I., Adams R.P. (2022). Polyploidy in Cupressaceae: Discovery of a new naturally occurring tetraploid, *Xanthocyparis vietnamensis*. J. Syst. Evol..

[51] Ronquist F., Teslenko M., Van der Mark P., Ayres D.L., Darling A., Höhna S., Huelsenbeck J.P. (2012). MrBayes 3.2: Efficient Bayesian phylogenetic inference and model choice across a large model space. Syst. Biol..

[52] Swofford D.L. (2002). PAUP*: Phylogenetic Analysis Using Parsimony (*and Other Methods).

